# Fertility preservation: is there a model for gender-dysphoric youth?

**DOI:** 10.3389/fendo.2025.1386716

**Published:** 2025-04-11

**Authors:** Michael K. Laidlaw, Jennifer Lahl, Angela Thompson

**Affiliations:** ^1^ Department of Endocrinology, Diabetes, and Metabolism, Michael K. Laidlaw, MD, Inc., Rocklin, CA, United States; ^2^ Department of Bioethics and Culture, Center for Bioethics & Culture Network, Pleasant Hill, CA, United States; ^3^ Department of Obstetrics and Gynecology, St. Joseph Hospital, Milwaukee, WI, United States

**Keywords:** reproduction, pediatrics, infertility, puberty, estrogen, testosterone, transgender

## Abstract

Assisted reproductive technologies (ART) and cryobiology advances over the past decades have offered hope to cancer patients who might not otherwise be able to have biological offspring due to the toxic nature of therapies that may lead to subfertility or infertility. Fertility preservation (FP) for youths with gender dysphoria (GD) poses an additional set of complications and obstacles because of the use of medications which block normal pubertal development such as gonadotropin-releasing hormone analogues (GnRHa) and medications which directly alter the genital tract such as cross sex hormones. Here we review the current state of knowledge and ethical concerns with FP focusing on issues when FP is used during adolescent and preadolescent reproductive development in the context of cancer and gender dysphoria treatment. Particularly for youths with GD, very little evidence-based research has been performed and much remains unknown with respect to long term harms to reproductive health and the ultimate success of FP and conception.

## 1 Introduction

Advances in cryobiology and assisted reproductive technologies (ART) over the past several decades have played a pivotal role in both human and animal preservation efforts. Our ability to cryopreserve gametes, embryos, and even gonadal tissue for extended periods of time has led to the preservation of exotic and endangered species. Furthermore, it offers hope to many cancer patients who are aware that the gonadotoxic drug therapies, essential for their treatment, might reduce their fertility or even render them infertile post-treatment (1). These technologies are also being advocated for use within treatment regimens for gender dysphoria in both adults and children. However, the use of puberty blocking medications, cross-sex hormones, and genital surgeries can adversely affect fertility and the ability to conceive.

When diagnosed with a bodily illness like cancer, for example, both adult men and women of reproductive age are often offered fertility preservation (FP). Females can decide to harvest ova, and males may decide to collect sperm for cryopreservation, or either sex might opt to create embryos for cryopreservation. In some instances, they may be offered to cryopreserve ovarian or testicular tissue. Cryopreserved ovarian and testicular tissue is then auto-transplanted back into the patient at a later time after treatment cessation to restore fertility. In females, the oocytes removed may undergo *in-vitro* maturation (IVM), undergo fertilization, and an embryo generated. However, the outcomes of these preservation options vary significantly, influenced by factors such as the success of cancer treatment, any subsequent surgeries (particularly for reproductive cancers like ovarian, cervical, and endometrial), the patient’s age, underlying health conditions, and the unfortunate reality that some might not recover and ultimately succumb to their illness. In the case of the demise of the patient, ethical concerns arise around the disposition of frozen gametes or embryos.

The efficacy of ART is still under scrutiny due to its relatively low success rates in achieving pregnancies that result in live births. Data from the Centers for Disease Control and Prevention for 2021 indicate that, in the United States, approximately 238,126 patients had 413,776 Assisted Reproduction Cycles (ART) cycles performed, which resulted in 91,906 live births—a 22% success rate of live births per cycle (2). Many factors influence *in vitro* fertilization (IVF) success rates, including the woman’s age, pre-existing fertility or other health conditions, the use of fresh versus frozen eggs in the IVF cycle, the use of fresh or frozen embryos, and whether donor eggs are used.

Here we will first discuss FP in cancer patients, followed by its use in gender-dysphoric patients in light of the effects of puberty blocking medications and cross sex hormones, and finally examine issues related to conception and ethics related to treatment.

## 2 Fertility preservation in cancer patients

### 2.1 Background

There are four methods that can be used to preserve female fertility for patients who have a cancer diagnosis and for whom recommended treatment is gonadotoxic. The methodologies have varying degrees of clinical success and are dependent on the maturity of the gametes at the time the techniques are utilized. The methods are: (1) controlled ovarian stimulation (COS) with generation of an embryo at the time of COS (the embryo is then frozen for implantation after treatment is rendered, should the patient desire this); (2) oocyte cryopreservation (a controlled ovarian stimulation is performed and instead of immediate fertilization to generate an embryo, instead, the retrieved oocyte is frozen so it may be used later on); (3) Ovarian tissue cryopreservation (a portion of the ovary is removed via surgery and then cryopreserved, with the goal of autotransplantation at a later time if feasible); (4) *In-vitro* maturation (IVM) of immature oocytes that are obtained pre-chemotherapy; the techniques for obtaining oocytes for this process vary significantly and the clinically meaningful live births achieved using this method are minimal.^1^ In male patients, there are two ways to preserve fertility. For male patients who have undergone spermarache (i.e., have reached sexual maturity, typically by Tanner stage 4), spermatozoa can be obtained via ejaculate or testicular sperm extraction and then frozen for later use to generate an embryo after fertilizing a mature oocyte. For male patients who have not yet reached sexual maturity (Tanner 1-3), testicular tissue can be removed via surgery and then cryopreserved with the goal of being able to autotransplant the testicular tissue later on, with the hopes for future *in vitro* maturation. This has not yet been shown in humans and as such is considered experimental [ (4), p. 135; 5, p. 1028]. Additionally, there is no way to ‘mature’ immature germ cells (spermatogonia) to mature spermatozoa outside of the testes.

### 2.2 Cryopreservation

Fertility preservation studies in adult women with cancer have shown that embryo cryopreservation has the highest pregnancy and live birth rate and therefore is considered the “gold standard,” yielding a live birth rate of 41% in this population (6). For the same population, using oocyte preservation results in a 50.2% pregnancy rate per successful cycle (excluding failed cycles) (7), and a live birth rate of 32% (6). Meanwhile, cancer patients who choose cryopreservation of their ovarian tissue have a 25% pregnancy rate, with live birth rate of 21% if IVF is utilized to achieve pregnancy; if ovarian tissue is cryopreserved and later autotransplanted and a pregnancy is achieved spontaneously, the live birth rate for this population is 33% (6). In 2019, the American Society of Reproductive Medicine (ASRM) cited studies in which individual centers reported their successful pregnancy and live birth rates, ranging from 29 to 33% for pregnancy rates and 23 to 25% for live birth rates (5). In 2017, Donnez and Dolmans (8) reported on 22 women who underwent ovarian tissue reimplantation after cryopreservation. They found a 41% pregnancy rate and a 36% live birth rate [ (8), p. 1661]. In 2022, Diaz et al. (9) published pregnancy rates after using various ovarian tissue cryopreserved methods followed by autotransplantation. The methods included strips, squares, and fragments of ovarian tissue, yielding pregnancy rates between 45-81% depending on the methods used. However, as the authors note, the review relied on authors who may be reporting only the successful outcomes and not the unsuccessful ones, and relied on small sample sizes–some involving only 4 patients [ (9), p. 12]. Critically, of all the reports on successful live births after ovarian tissue cryopreservation and subsequent auto-transplantation, only two live births occurred when the ovarian tissue was cryopreserved in childhood (premenarche). That is, the vast majority of the successful live births occurred when ovarian tissue was cryopreserved after the pubertal menarchal transition (Tanner stage 4 and above). This will be discussed in detail later in the report.

While cryopreservation of ova or ovarian tissue was once considered experimental, the American Society of Reproductive Medicine (ASRM) removed the experimental label for oocytes in 2012 and for ovarian tissue in 2019 (5, 10). While the ASRM did not delineate specifically between adult and pediatric females when they removed the experimental label to these procedures, they note “given that this [pediatric population] is a particularly vulnerable population, careful counseling and informed consent are especially recommended” and the only option for prepubertal girls to cryopreserve gametes is via ovarian tissue cryopreservation [ (5), p. 1027]. Critically, researchers and pediatric fertility specialists note “the pediatric population is vastly underrepresented in the clinical studies and data used to generate the ASRM guidelines” and state more research is still required to inform improved care for female pediatric patients (10). Special consideration should be given to pediatric females undergoing OTC to ensure the procedure stays within IRB-approved research parameters, especially given the limited data in this unique population. ASRM acknowledges that for pediatric cancer patients of both sexes, fertility preservation may be offered—if time permits before the urgent initiation of gonadotoxic drug therapy or irradiation—especially when the treatment plan poses a significant risk to fertility.

Cancer patients often express concerns about the potential risks to their future offspring subsequently born, including cancer or other abnormalities. Yet survivors of childhood cancer should be aware that data indicates there are no increased risks of cancer for offspring born later (11). However, limited studies suggest that children born via ART, such as *in vitro* fertilization, may face higher risks of poor perinatal outcome, birth defects, and epigenetic disorders (12). The American College of Obstetricians and Gynecologists (ACOG) recognizes perinatal risks associated with ART, especially the risk of multifetal gestation. This can increase maternal and fetal morbidity and mortality due to heightened risks of preterm birth and preeclampsia (13, 14). Regardless of fetal number, pregnancies achieved through ART have been linked with increased risk of preterm birth, low birth weight, perinatal mortality, cesarean delivery, placenta previa, placenta abruption, preeclampsia, vasa previa, stillbirth, and severe maternal morbidity (as measured by increase in blood transfusion). Recent data suggest the use of ART to achieve pregnancy may be associated with an elevated risk of certain pediatric cancers. However, these data are limited and no definitive conclusions can be drawn (15).

Boys offered FP in prepubescence and early puberty (Tanner stages 1 and 2, in which gametes have not yet matured), have only the option of testicular tissue preservation available to them, since they have not yet developed mature spermatozoa (5, 16). As mentioned earlier, in male children this is still considered experimental. Girls offered FP in prepubesence and early puberty have ovarian tissue preservation and *in vitro* maturation as possibilities. In female children, while technically feasible in theory, data for pediatric patients are scarce. Ovarian tissue cryopreservation (OTC) has been used in mostly Western European centers around the world since the late 1990s. However, obtaining precise data on the number of OTC procedures performed is challenging since only successful outcomes are reported in the literature.

Donnez (8) reported 130 live births in 2017 from females who underwent OTC followed by Ovarian Tissue Transplant (OTT). Diaz (9) later reported up to 140 such births in 2022. It is important to remember that only successful live birth outcomes are reported, so the true denominator of females who undergo this procedure remains unknown [ (8), see p. 1662]. This makes determining the procedure’s success rate in achieving live births difficult. One study (17) reported outcomes of transplanted ovarian tissue in Denmark from 2003 to 2014, noting that 41 women underwent this procedure. The mean age of adult women who underwent the procedure was nearly 33 years. Autotransplanted ovarian tissue survived up to 1-4 years in over half of these patients. Other studies have reported longer duration of ovarian tissue function, with the duration depending on several factors. Among these factors is the technique used for ovarian cryopreservation, which currently is not standardized (18). The true success rates for OTC/OTT remain elusive, since the denominator of who undergoes these procedures is unknown (8), and the techniques by which OTC is performed are not standardized [ (9), p. 12].

The first documented case of FP using OTC in a minor female patient was reported in 2007 (19). With prepubescent and early pubertal girls, immature oocytes could be retrieved from the ovarian tissue, matured *in vitro*, then cryopreserved for use in future fertility treatments (20). The older pediatric patient (late stage to post-puberty) would have ova and sperm cryopreservation available to them (21).

There have been only two patients reported in the literature for whom live births were achieved after OTC was performed as premenarchal children, and then ovarian tissue was autotransplanted years later. The case reports consist of a female child age 13, noted to be premenarchal at the time of OTC, which was performed prior to stem cell transplant for sickle cell anemia. She had the ovarian tissue transplanted (OTT) back into the pelvis 10 years after cryopreservation and underwent resumption of ovarian tissue function and conceived without use of ART and delivered a liveborn neonate (22). Another patient was age 9 at the time of OTC and underwent OTC prior to treatment for beta thalassemia. She also had OTT performed later on after her treatment was completed and pregnancy was achieved (23). A third patient was aged 14 at the time of OTC however the authors did not mention in their research if she had undergone menarche yet. She required two courses of autotransplantation of thawed ovarian tissue, the first of which did not result in a live birth after 4 rounds of IVF; the second autotransplanted tissue was a success in that IVF was performed and an embryo was generated and able to be transplanted, resulting in a preterm live birth (35 weeks), with another pregnancy undelivered at the time of the report in 2021 (24).

Testicular tissue cryopreservation for the prepubertal and early pubertal stage male remains experimental (ASRM 2019). There has been no success in maturing spermatozoa from spermatogonia *in-vitro* for the human male (25). Animal models, on the other hand, have shown successful *in vitro* maturation of autotransplanted testicular tissue even with xenotransplantation; however, this technique has not yet been shown to work in humans^^2^^.

### 2.3 *In vitro* maturation

*In vitro* maturation refers to maturing immature oocytes outside of the ovary. There are significant limitations with this technique that preclude its use in female children and adolescents who have not yet achieved menarche (Tanner 1-3), which will be explained later in this section. IVM is a process by which immature oocytes are retrieved from the ovaries and are matured outside of the body. Its technology has been known and used for decades in animal breeding programs but use in humans has not been well developed due to technological advances in controlled ovarian stimulation (COS) protocols developing concomitantly (27). Consequently, it has been hampered by varying protocols and low numbers of successful live births. The first live human birth using IVM was documented in 1991 (28). To date, IVM is used in adult female patients in whom the risk of life-threatening ovarian hyperstimulation syndrome (OHSS) is unacceptably high, such as patients with polycystic ovary syndrome (PCOS).

A significant barrier to IVM success is the importance of maintaining the ovarian somatic cells necessary to help the developing oocyte mature. These are called cumulus complex cells (granulosa cells are within this complex). At the time of birth, female infants will have all the oocytes they will ever have. The immature oocytes are ‘arrested’ in the diplotene phase of meiosis I (29, 30). A flat, single layer of granulosa cells surround each immature oocyte; the immature oocyte with its layer of surrounding granulosa cells is called the primordial follicle. The number of primordial follicles are in the millions during gestation and at birth only about a million remain. The primordial follicles undergo a programmed cell death or remain in a quiescent period throughout childhood until pubertal transition of menarche is reached at Tanner stage 4. Once GnRH is released in a pulsatile fashion to signal to the anterior pituitary to release FSH and LH, the cascade of physiologic response at the level of the ovary begins and ovulation can occur. By this time, only about 400,000 primordial follicles remain. The primordial follicle destined to ovulate is ‘rescued’ from apoptosis and grows in size in response to FSH. Crucially, the supporting granulosa cells are also changed in response to FSH from flat to cuboidal and intricate signaling connections are maintained between the supporting cells and the oocyte which are crucial to sustain the developing follicle. Under the continued influence of FSH, the dominant follicle continues to grow, surrounded by fluid present in the spaces between the surrounding granulosa cells called the cumulus oophorus. Theca cells adjacent to the granulosa cells respond to LH which secrete androstenedione and testosterone, which is aromatized to estradiol by the granulosa cells. The estradiol increase is then communicated to the pituitary and causes a surge in LH release which leads to ovulation (31).

In females who have already undergone menarche, the ovaries can be monitored via ultrasound to determine which oocyte is likely to become the dominant follicle by measuring its size during the follicular phase of the ovulatory cycle. The cumulus cells can be preserved with the oocyte during retrieval and sustained during the process of IVM. However, in premenarchal females who do not have mature cumulus cells at the time of oocyte retrieval, this is a significant limitation, as there is no ability to use any kind of ‘mild stimulation’ with gonadotropins/hCG prior to oocyte retrieval. As of 2021, a review of IVM retrieved from immature ovarian tissue (known as monophasic IVM) showed that in adult females, 5 live births were achieved out of 512 patients identified as undergoing this technology in a series of case reports, cohort, observational, and cross sectional studies [ (32), see pp. 3-6, **Table 1**].

**Table 1:** Clinical Case Scenario.

Case Scenario
To illustrate further, let us take the hypothetical example of a Tanner stage 2 natal female who received GnRHa beginning at age 11 and then continued to have further pubertal development blocked through age 14. The patient was then dosed with high dose testosterone at age 14 with a dose schedule according to the Endocrine Society Guideline (31). The patient never received FP. At the age of 21, the patient considers pregnancy. If the patient seeks a natural pregnancy, then the patient is in the difficult position of needing to discontinue testosterone and then to advance through the remaining stages of female puberty, if that is even possible. There are multiple unknowns in this scenario. Questions include: 1) Will the pituitary gonadal axis recover from GnRHa followed by testosterone, allowing for the potential resumption of natural female pubertal development? 2) Will the ovaries respond to LH and FSH signaling appropriately? 3) Will normal ovulation and menstruation develop? 4) Will a fertilized ovum be able to implant normally? 5) Will a baby be able to develop normally and come to term given the likely extensive histologic changes to the reproductive tract? 6) What types of maternal or fetal complications might develop during pregnancy given this scenario?

In regard to fertility preservation, premenarchal children and adolescents (Tanner 1-3) are those in whom the ‘monophasic’ IVM data would apply, as the ovaries in these patients have not yet matured to obtain cumulus complexes. There have been no known live births from IVM of ovarian tissue obtained in premenarchal female children. The ASRM in 2021 cites “the chance of a single immature oocyte resulting in a live birth is only 1.1%” using currently available IVM techniques [ (3), p. 299]. Indeed, a cautionary note from the authors in one review states that “it has been hypothesized that prepubertal ovaries need a maturation phase to obtain optimal follicle function, which would imply the impossibility of harvesting COC (cumulus oophorus complexes) ex vivo or, if COC could be obtained, whether these would present with lower maturation capacity (18-33%) [ (32), p. 11]. To further illustrate the inability of minors to utilize IVM technique for fertility preservation, the ASRM states that intracytoplasmic sperm injection (ICSI) is combined with the use of IVM and does *not recommend the use of vitrification of immature oocytes* owing to reports of poor outcomes. What this means is that in order to utilize IVM, the oocyte that is retrieved must then be immediately fertilized [ (3), p. 301]. This is feasible in an adult female who is able to undergo bi-phasic (also known as follicular priming) IVM, as an adult female has the capacity to understand and consent to the generation of an embryo. However, it is ethically impermissible to potentially utilize this technology in a minor. Thus, the use of IVM after ovarian retrieval in a prepubertal or early pubertal (Tanner 2/3) adolescent cannot be reliably utilized as a means for fertility preservation in this cohort.

### 2.4 Fertility preservation networks

Fertility preservation within the context of oncology care has figured saliently into treatment plans for several years; however, for families of pediatric patients these discussions have still shown to be lacking (16). Importantly, fertility preservation networks within and between oncology care centers have been established (33). However, these networks also have ‘human factor’ barriers to implementation which can undermine effectiveness [ (33), see p. 4, Table 2]. Oncofertility preservation networks are also distinguished by the fact that they exist to preserve fertility for patients in whom malignancy exists, and thus their preserved gametes carry risks for malignant potential; the patient in whom fertility was protected is the only recipient for their gametes. This is a key difference from fertility preservation for children and adolescents who are otherwise physically healthy, in that their reproductive cells are being preserved for not only themselves, but for perhaps others to use. Ethically, this brings up a host of questions regarding third-party reproduction, as discussed here.

## 3 Fertility preservation in gender-dysphoric youth receiving GnRHa and cross sex hormones in early pubertal development

### 3.1 Background

Fertility preservation in the pediatric population has expanded beyond those diagnosed with childhood cancers (34). Even though procedures to preserve fertility are available for gender-dysphoric youth undergoing gender affirmative therapy (GAT),^3^ some studies in North America show that less than 5% of adolescents receiving GAT even attempt fertility preservation (FP) (36, 37). Discussing the ethics of FP in this population, Harris et al. explain that this is very concerning as “up to 95% of transgender children undergoing medical treatment could experience permanent sterility” [ (38), p. 2454]. Another study (39) from Australia showed that out of 53 natal males offered FP, 62% chose FP compared to 49 who were natal females, none of whom chose fertility preservation. This large mismatch in FP between natal females and natal males occurred perhaps because collecting ova or ovarian tissue in natal females is more onerous than collecting sperm in natal males. Nonetheless all of these FP studies are concerning because, as Harris et al. note, “transgender adults regret not being able to have biological children” [ (38), p. 2454].

Fertility preservation counseling in young people undergoing gonadotropin-releasing hormone agonist (GnRHa), also known as “puberty blockers,” followed by masculinizing or feminizing hormones, is not standardized, and a wide range of utilization of FP counseling has been reported. In a systematic review, Stolk et al. report studies varying from as low as 13.5% undergoing FP counseling to up to 100% of patients undergoing fertility preservation counseling [ (40), see pp. 9-10, Table 3]. For those who actually utilized fertility preservation techniques after counseling, the data ranged from 0-62%. Data are not standardized and highly variable in this area of fertility preservation counseling as well as in the utilization of the actual techniques. This calls into question just how well informed consent discussions convey the actual risks for invasive and fertility-risking hormonal interventions as well as the outcomes given the limited success of currently available FP techniques.

### 3.2 Tanner stages of sexual development and their relation to fertility

It is extremely important to recognize that fertility preservation techniques, and their subsequent outcomes of successful live births after their utilization, depend greatly on the stage of pubertal development at which such techniques are utilized.

Sexual development is divided into 5 stages called Tanner stages. Stage 1 is the prepubertal state before pubertal development of the child begins. Stage 5 is full adult sexual maturity. Stages 2 through 4 are various phases of pubertal development (41). Awareness of the Tanner stage of the developing adolescent is useful to assess for maturation of sex organ development leading to fertility. For girls, the first menstruation (menarche) occurs about two years after Tanner stage 2 and will typically be at Tanner stage 4 or possibly 3 (42). The first appearance of sperm (spermarche) will typically be Tanner stages 4. If puberty is blocked before reaching these critical stages, then the sex glands will be locked in a premature state and incapable of natural fertility.

Fertility preservation techniques for pubertal stages prior to maturation of the gametes (occurring around menarche in females and spermarche in males) are very limited in the pediatric population at Tanner stage 1 and the pre-gamete maturity developmental stage of Tanner 2. The Endocrine Society’s clinical practice guideline (35) recommends beginning puberty blockers as early as Tanner stage 2, which is the very beginning of puberty. Gametes are not mature in patients of either sex at Tanner stage 2 of pubertal development (42, 43). One can see that if the developing person’s puberty is blocked at Tanner stage 2 or 3, as advocated by the Endocrine Society’s guidelines, this is prior to becoming fertile. The gonads will remain in an immature, undeveloped state. This dramatically reduces fertility preservation options and success. As already discussed, for females, potential options are limited to ovarian tissue cryopreservation (OTC) and IVM. With respect to natal females who have been treated with GnRHa in early puberty followed by testosterone as a part of gender affirmative therapy, there have been no reports of live births after OTC/OTT.

In human males, testicular tissue cryopreservation (TTC) is the only option for prepubertal or early pubertal boys (prior to spermarche) to preserve fertility prior to the administration of GnRHa and estrogen. For cancer patients, TTC is performed in order to later attempt to mature immature spermatogonia from the cryopreserved testicular tissue, either by *in-vitro* methods or by autotransplantation into the male later on in adulthood after treatment with the gonadotoxic agents is complete. This process remains experimental, as it has never been demonstrated in humans that maturation of immature spermatogonia into haploid spermatozoa is able to occur either *in vitro* or via auto-transplantation of cryopreserved immature testicular tissue (16, 44). The only live birth that has been achieved using these techniques was in a non-human primate (45). Human spermatozoa have not been obtained from immature testicular transplantation nor *in vitro*, and therefore their fertilization capacity is also unknown. Also unknown is the genetic and epigenetic integrity of spermatozoa generated from non-human primates; the only data we have at this point is the non-human primate offspring with ‘normal’ developmental behavioral testing for this animal.

### 3.3 Negative effects of cross-sex hormones on the reproductive tract

The supraphysiologic doses of testosterone used in GAT are very high, on the order of 6-100 times above the normal reference range (46). The use of supraphysiologic doses of testosterone leads to substantial histologic changes to the natal female reproductive system. For example, in a review of 11 histopathologic studies of resected ovaries of trans males receiving testosterone, 34.9% had polycystic-appearing ovaries and 0.7% had benign ovarian neoplasms (47). Endometrial findings after hysterectomy showed that 48.7% were atrophic with 46.6% proliferative and 3.0% secretory. Another study (48) of natal females administered testosterone in GAT, aged 18-56, found that 57% of 35 ovariectomy cases showed multiple bilateral cystic follicles and 80% of 40 uterine resections displayed endometrial stromal fibrosis.

The risks of supraphysiologic testosterone to the healthy physiological functioning in females are being elucidated as more patients present with exposure to high androgen levels for prolonged periods of time. Testosterone is aromatized to estrogen, and while high doses of testosterone will suppress the hypothalamic-pituitary-gonadal axis leading to clinical estrogen deficiency, eventually some fraction of excess testosterone will be aromatized to estrogen (49). There are concerns that this increase in unopposed estrogen will lead to increased risks of female gynecologic cancers in natal female transgender men who take such supraphysiologic doses of testosterone. Mueller and Gooren have noted, as far back as 2008, that “an unresolved question is whether in the long term the administration of cross-sex hormones is safe, at least as safe as administration of sex steroids to a subject receiving long-term sex-appropriate sex steroids … [C]omplications occurring in the longer term are often seen in general practice … only occasionally reported in the scientific literature. So, it is likely that there is an underreporting of (serious) complications of cross-sex hormone therapy” [(50), p. 197].

The effect of supraphysiologic doses of exogenous androgens on female fertility is not well characterized. Pregnancy is possible for females exposed to supraphysiologic testosterone for varying lengths of time, and cases have been reported. However, to our knowledge, there are no data of pregnancies occurring for any young females who, prior to Tanner stage 4, had ovarian tissue cryopreservation performed prior to GnRH analogs, followed directly by supraphysiologic doses of testosterone, under the auspices of GAT.

In a study of 214 natal male patients, grouped into Tanner stages of development, who had undergone hormonal intervention with estrogen to affirm their gender identity, orchiectomy specimens were stratified according to Tanner stage and use of GnRHa and cross-sex hormones (43). The data were reported only for Tanner stages of development, and whether or not individuals were “adolescents” or “adults”; but the specific ages of the study participants was not reported. It is important to recognize that normal Tanner stage 2 of development can be as young as age 9 in male children (42). In the de Nie study (43), only 4.7% of patients had full spermatogenesis, however all 10 of these patients had initiated GAT at Tanner stage 4 or higher. A complete absence of germ cells was found in 7% of subjects, all of whom had begun GAT in adulthood. With respect to those who had normal puberty blocked with GnRHa and then proceeded to have cross-sex estrogen administered while at Tanner stage 2 or 3, all samples showed immature germ cells with spermatogonia being the most common type of gamete. These data provide the first ever confirmation that blocking natal male sex puberty at Tanner stage 2 and proceeding directly to cross-sex estrogen use precludes gamete maturation. There is also no ability at present to initiate and complete spermatogenesis from spermatogonia “*in vitro*” or via autotransplantation of testicular cryopreserved tissue in humans—it is experimental. Thus, any reference to fertility preservation in young male children and adolescents in early puberty, prior to undergoing hormonal intervention to affirm a ‘female’ gender identity, is an exercise in theory only.

### 3.4 Special considerations for fertility preservation in natal females and males beginning GnRHa at Tanner 2/3 pubertal development

Little, if any, data exist specifically regarding natal females who have had puberty blocked at an early Tanner stage (2 or 3) and then have gone on to receive supraphysiologic testosterone as part of gender affirming hormone intervention. Therefore, the long term effects of hyperandrogenism on the immature female reproductive tract are unknown. The possibility that high dose testosterone is permanently damaging to immature ovaries cannot be excluded.

The considerations for fertility preservation for these premenarcheal patients who undergo gender affirmative therapy are more complex than for cancer patients. This is because in the GAT population (1) the long-term effects of GnRHa on the female reproductive tract and pituitary gonadal axis are unknown, (2) the long-term effects of testosterone on ultimate fertility are unknown, (3) ovarian tissue autotransplantation cannot be attempted while the patient takes testosterone (as normal pituitary gonadal function is inhibited), (4) the patient could choose to refrain from testosterone therapy for several months or longer prior to OTT; however, multiple uncertainties exist as to whether and for how long the tissue would be viable—and if ovulation would occur. A recent review of ovarian stimulation and cryopreservation in natal females including transgender men under age 18 concludes: “While it is considered standard practice in adults, long term outcomes are largely unknown in the young and the procedure should be considered experimental in prepuberal and premenarchal patients” [ (51), p. 11].

Other possibilities for fertility for the patient blocked in early puberty (Tanner 2/3) would include resection of ovarian tissue prior to beginning GnRHa, similar to the harvesting of ovarian tissue in cancer patients (ovarian tissue cryopreservation). This would avoid histologic changes to the ovaries induced by testosterone. However, the uncertainties about the resumption of normal pituitary gonadal axis and reproductive tract function are similar to the above.

Likewise, the considerations of fertility preservation for Tanner 2/3 natal male patients who undergo gender affirmative therapy are more complex than for cancer patients. This is because (1) the long-term effects of GnRHa on the male reproductive tract with respect to future fertility are unknown, (2) the long-term effects of high dose estrogen on fertility and sexual function in this population are unknown, (3) it is not known if it is possible to stop GnRHa and estrogen in order to allow the male to continue through natural puberty and allow for normal spermatogenesis for sperm harvesting. More importantly, as stated previously, the use of the patient’s own cryopreserved testicular tissue for *in vitro* spermatogenesis has been unsuccessful in humans, so far, so that is not a possibility (52).

Fertility preservation for children is costly, remains experimental, is invasive, is not without risks, and offers no guarantee of genetic offspring later in life (53).

### 3.5 Sexual dysfunction

Another problem with blocking puberty at an early stage is sexual dysfunction. The child will continue a chronological age progression toward adulthood—and yet remain with undeveloped genitalia. This will lead to sexual dysfunction, including erectile dysfunction and potential inability to orgasm and ejaculate for the natal male. For the natal female with undeveloped genitalia, the types of sexual dysfunction may include vaginal dryness with potential for vaginal wall abrasion, painful intercourse, and impairment of or pain with orgasm. A recent study (54) demonstrates that the likelihood of vaginal lacerations requiring repair are increased in transgender and nonbinary natal female patients undergoing minimally invasive hysterectomy for GAT after they had been on preoperative testosterone an average of 2.5 years, compared to similar patient demographics who were not exposed to preoperative testosterone use.

## 4 Conception and pregnancy

A natal male who has cryopreserved sperm would need to be in a partnered relationship with a woman who has an intact uterus and could carry the pregnancy to term. Methods of fertilization include either intrauterine insemination for *in vivo* fertilization, or the harvesting of ova, allowing for embryos to be generated in the laboratory and then transferred back into the woman’s uterus. If not in a partnered relationship, there would be the need to employ the use of a surrogate. If gestational surrogacy is chosen, then donor ova would be used to create embryos for transfer. If traditional surrogacy is used to achieve pregnancy, the surrogate mother would provide her own ova and could either be artificially inseminated or have ova extracted for IVF to create embryos for uterine transfer.

Trying to conceive in the natal female population which has transitioned after menarche will look different, depending on the patient having had surgery on their reproductive tract or not. If a healthy uterus is intact, then a surrogate may not be necessary. If the patient is in a male partnered relationship, donor sperm would not necessarily be needed. If the patient is a trans-identified natal female partnered with a natal female then sperm donation would be necessary to create embryos via IVF. The embryos(s) would then be transferred into the partner with an intact, healthy uterus capable of giving birth or a surrogate. What about the known effects of elevated serum testosterone on the pregnant patient as well as the growing fetus? We do know that women with androgen excess, such as polycystic ovarian syndrome (PCOS), have clinically increased risk for adverse pregnancy outcomes (55, 56). Valdimarsdottir et al. (57) measured androgens in pregnant women with and without PCOS. They found that having PCOS with the highest testosterone levels in pregnancy (>2.36 nmol/L, normal range 1.44-2.36 nmol/L, converted to 41-68 ng/dL) is associated with increased risk of pre-eclampsia in pregnant women with PCOS, but not gestational diabetes, gestational hypertension, or adverse effects on birth weight. However, data are mixed in regards to greater risk of preterm birth, perinatal mortality, congenital abnormalities, metabolic disorders, diseases of the nervous system, as studies have demonstrated an increased risk of these complications as well, and, because of these complications, patients are more likely to deliver by cesarean birth (58).

The measured androgen levels in pregnant women with PCOS have not been reported to contribute to clinically observed virilization of the female infant at the time of birth (59). This is due to the fact that, in pregnancy, women and female fetuses are protected from the increased concentrations of androgens by enhanced binding to sex hormone binding globulin, competition by progestins for binding to the androgen receptor, disposition of androgens to more biologically potent compounds, and placenta aromatization of androgens [ (60), p. 1052]. Despite these protective mechanisms, androgen excess can occur in pregnancy, as manifested by maternal hirsutism and virilization resulting from ovarian disease or iatrogenic insult. It is important to recognize that the androgen levels in pregnant women with PCOS are much lower than that of those in females undergoing masculinization hormone administration (levels achieved are to a goal of at least 320 ng/dL and up to 1000 ng/dL, normal male sex testosterone levels) (35); it can thus be demonstrated that the doses of exogenous testosterone administered during GAT can be considered an iatrogenic insult in pregnancy. Indeed, there is little scientific literature describing pregnancy experiences among transgender men or the effects of exogenous administration of testosterone on fertility, pregnancy, and neonatal outcomes [ (61), p. 1120]. Due to these risks and complications, it is advised that transgender men do not take testosterone while attempting to conceive or through the duration of the pregnancy (62, 63).

The female fetus can be affected by elevated circulating maternal androgens as early as 7 weeks, during differentiation of the external genitalia. Exposure to excess androgens can result in partial or complete labia fusion and clitoromegaly (which can also occur after 12 weeks gestation). If there is enough circulating androgen to cause maternal virilization (as clinically noted by hirsutism) in the pregnancy, then the female fetus also is at risk for virilization, although it is important to recognize that virilization of the female fetus can also be unaccompanied by maternal virilization [ (60), p. 1053]. The amount of virilization depends on the amount of androgen, end organ sensitivity, and degree of aromatization by the placenta to non androgenic steroids. The two major causes of maternal endogenous gestational hyperandrogenism are luteomas and ovarian cysts called theca-lutein cysts, as well as placental aromatase deficiency, cytochrome P450 oxidoreductase deficiency (a form of congenital adrenal hyperplasia), PCOS patients with insulin resistance, Sertoli Leydig tumors, Krukenberg tumors, Brenner tumors, fibrothecomas, mucinous and serous cystadenocarcinoma, dermoid cysts, and adrenal tumors.

The existing literature on the fertility in women with endogenous hyperandrogenism is limited, and literature on the effects of exogenous testosterone administration in the female are also very limited, with more questions than answers (62). Adverse events and effects of long-term testosterone exposure on ovarian tissue are still under study (64). There have been no prospective studies to date evaluating the effect of long-term hormone therapy on fertility, although there are a few reports in the literature of transgender men who have carried pregnancies and given birth (62). The total number of transgender men who have attempted pregnancy is unknown, and fecundity rates cannot be calculated. One study described in the review by Moravek (62) showed that 80% had resumption of menses within 6 months of stopping testosterone, but most had been on testosterone for under 2 years. The reproductive health of offspring resulting from testosterone exposed oocytes, without direct intrauterine exposure, is unknown. Evidence suggests that offspring from women with PCOS may have impaired fertility although these outcomes have not been studied in any offspring of testosterone treated transgender men. Interestingly, Light et al. (61) reported a change in gender dysphoria during pregnancy, with some participants reporting improvements in gender dysphoria, with a new sense of connection to their bodies. As one patient described, “It was relieving to feel comfortable in the body I’d been born with” [ (61), p. 1123]. Other patients, however, reported feeling an increase in dysphoria during pregnancy.

If ART is desired or required to achieve pregnancy in natal females who have been exposed to exogenous testosterone in GAT, outcomes are extremely limited. For those who do not want or are unable to discontinue testosterone, then the sequence of ovarian tissue cryopreservation followed by *in vitro* maturation of the oocyte from small antral follicles, followed by *in vitro* fertilization has been performed in three studies. In two of the three studies, there were no blastocysts generated, and between 34%-38% of mature oocytes were generated *in vitro*. The only study that showed generation of a single blastocyst using this method had over 1,900 cumulus oophorus complexes (COS) generated, with an ovarian maturation rate of 23.8% [ (40), see pp. 20-21, Table 7]. There have been no live births from this method, and an extremely low generation of day 5 blastocysts from COS. For those who undergo ovarian tissue cryopreservation with autologous ovarian tissue transplantation, no data exist regarding this technique specifically in transgender men—only for natal females who have not been taking either GnRHa or exogenous testosterone. For those who prefer or who require ART using endogenous ovarian maturation/stimulation protocols to generate cumulus oophorus complexes (COS), for use in either ovarian cryopreservation or immediate embryo generation/cryopreservation, data regarding the optimal management of testosterone administered around ovarian stimulation are limited to anecdotal reports from clinics that require testosterone discontinuation for 1-6 months prior to ovarian stimulation (62), or small retrospective studies (40), some of which included natal females who had not been on exogenous testosterone, or only GnRHa, but not both, and not administered at Tanner stage 2 of pubertal development. Overall, ovarian studies in natal females treated with exogenous testosterone have yielded conflicting results regarding the association of long-term testosterone with polycystic ovarian morphology on ovarian histopathology, and there are only limited data on fertilization or embryogenesis from oocytes previously exposed to testosterone (62).

## 5 Conclusion

Fertility preservation in pediatric populations prior to gamete maturation is experimental, with limited data on reproductive success as adults. Fertility preservation in pediatric populations prior to gamete maturation is a nascent technology. In the pediatric male population it remains experimental. In females, reproductive success is extremely limited when utilized prior to gamete maturation. At present, the ethical framework in which to utilize these experimental technologies in pediatric patients are for children who have a physical locus of disease, such as cancer, and require chemotherapy or radiotherapy to preserve their lives (65, 66). These children have no other option for preserving their fertility. The dire nature of their conditions does not allow them to wait for pubertal maturation before beginning cancer therapy, as waiting will have detrimental effects on their ultimate survival. A systematic review of the literature on offering FP to gender-dysphoric youth shows that FP is currently not the standard practice. There are many barriers, such as the lack of knowledge among healthcare providers around FP, which impedes counseling and discussion with patients and their parents. Currently, there remains a paucity of data in the literature about the effects of GAT on reproductive organs (67).

Children and adolescents who identify as transgender do not have a physical locus of a life-threatening diagnosis, such as cancer or other imminent danger to their reproductive tracts. Therefore, iatrogenically causing impaired fertility with GnRHa and cross-sex hormones removes from these children their right to an open future to decide their fertility goals. It is unethical to induce infertility/subfertility in children and young adolescents under the auspices of GAT, and then offer experimental, invasive, nascent fertility preservation in children as a way in which to circumvent this iatrogenesis.

The counseling process regarding fertility preservation in gender-dysphoric youth is new and not standardized, and parents themselves do not necessarily understand these processes—even when their children have cancer. General family satisfaction with the process of FP counseling is lacking; in one study, “only 30% of parents were satisfied with the FP counseling they received regarding their children,” [(16), p. 11]. These data are highly concerning regarding the way FP options are actually discussed with patients and families when a child has a cancer diagnosis. Therefore, it would seem even more concerning in FP counseling for transgender-identifying children and adolescents, given the relatively unknown repercussions of combining GnRHa, cross-sex hormones, and potential surgeries.

Certainly, a discussion of fertility preservation with the child and parents is important to be had; however, children—and often even adolescents—lack the foundational understanding of biology and reproduction necessary to understand advanced fertility preservation techniques, particularly in the context of GAT. Likewise, parents, who are generally a part of the informed consent process, would be expected in many cases to have difficulties comprehending the complex biological and ethical aspects of FP in relation to GAT.

Children and young adults also lack the maturity and life experience to fully appreciate what it means to beget genetic offspring. Furthermore, children and adolescents do not have the capacity to understand the physical realities of pregnancy—including the potential of risks that hormonal interventions in early adolescent development portend. Indeed, even adults have difficulties in understanding these potential risks. How could an eleven-year-old natal female child understand the ramifications of a uterus exposed to GnRH analogs, followed by supraphysiologic doses of testosterone, and the risks that this could have to gestation in adulthood? We currently have no information regarding pregnancy outcomes in patients exposed to this regimen. Also, it is known that testosterone administered at doses consistent with GAT increases systolic blood pressure (68). But does this lead to chronic hypertension, and will it then lead to the well-known risks that chronic hypertension has to pregnancy and perinatal outcomes? Again, we have no data.

Given the challenging nature of fertility preservation for pediatric patients in early puberty, the relatively little data available for gender-dysphoric youth treated by GAT and opting for FP, and the dearth of any data regarding children born via assisted reproduction from ovarian gonadal tissue preservation and/or ova and sperm freezing, we should not be advancing the current model of FP to this patient population or their parents as a best practice for having biological children in the future. After years of promoting medical interventions for transgender-identifying patients, the scientific and medical community has started acknowledging the significant limitations and risks of the current ability to ‘preserve fertility’ in these patients. In a recent paper applying a ‘gender-affirming approach,’ Powers et al. expressly state: “If the patient desires to have children or undergo fertility care in the near future, they may consider delaying or ceasing GAHT [gender-affirming hormone therapy] until after the fertility care is complete” [ (69), p. 7]. Identifying as transgender or gender-diverse is not a pathological physical condition. Therefore, the medical establishment does not have the right to introduce iatrogenic medical risks to these children and adolescents that compromise their healthy physiologic function.

## Footnotes

1 The ASRM removed the experimental label for IVM in 2021, however this technique has significant challenges in early puberty as will be discussed3].

2 There has been a single report out of the People’s Republic of China in which in-vitro testicular organogenesis from human fetal gonads was able to produce fertilization-competent spermatids. However, the study was ethically fraught, generating a two-cell blastocyst using aborted male fetal testicular tissue to fertilize donated oocytes. In vitro testicular organogenesis from human fetal gonads produces fertilization-competent spermatids [26].

3
*Gender affirmative therapy (GAT)* refers to the medical and surgical treatment of the psychological condition of gender dysphoria consisting of puberty-blocking medications, such as gonadotropin-releasing hormone agonists and cross-sex hormones as well as surgical modification of the breasts and genitalia, as described in the Endocrine Society’s “Endocrine Treatment of Gender-Dysphoric/Gender-Incongruent Persons: An Endocrine Society Clinical Practice Guideline” [35].
